# Pyrethroid resistance persists after ten years without usage against *Aedes aegypti* in governmental campaigns: Lessons from São Paulo State, Brazil

**DOI:** 10.1371/journal.pntd.0006390

**Published:** 2018-03-30

**Authors:** Maria de Lourdes Macoris, Ademir Jesus Martins, Maria Teresa Macoris Andrighetti, José Bento Pereira Lima, Denise Valle

**Affiliations:** 1 Laboratório de Entomologia Aplicada, Sucen, Marília, SP. Brazil; 2 Laboratório de Fisiologia e Controle de Artrópodes Vetores, Instituto Oswaldo Cruz, Fiocruz. Rio de Janeiro, RJ. Brazil; 3 Instituto Nacional de Ciência e Tecnologia em Entomologia Molecular (INCT-EM), Rio de Janeiro, Brazil; 4 Laboratório de Biologia Molecular de Flavivírus, Instituto Oswaldo Cruz, Fiocruz. Rio de Janeiro, RJ. Brazil; Faculty of Science, Mahidol University, THAILAND

## Abstract

**Background:**

*Aedes aegypti*, vector of dengue, chikungunya and Zika viruses, is found at high densities in tropical urban areas. The dissemination of this vector is partially the consequence of failures in current vector control methods, still mainly relying upon insecticides. In the State of São Paulo (SP), Brazil, public health managers employed pyrethroids against *Ae*. *aegypti* adults from 1989 to 2000, when a robust insecticide resistance monitoring system detected resistance to pyrethroids in several *Ae*. *aegypti* populations. However, pyrethroids are also the preferred compounds engaged in household applications due to their rapid *knockdown* effect, lower toxicity to mammals and less irritating smell.

**Methodology/Principal findings:**

We evaluated pyrethroid resistance in *Ae*. *aegypti* populations over the course of a decade, from 2004 to 2015, after interruption of pyrethroid public applications in SP. Qualitative bioassays with papers impregnated with a deltamethrin diagnostic dose (DD) performed with insects from seven SP municipalities and evaluated yearly from 2006 to 2014, detected resistance in most of the cases. Quantitative bioassays were also carried out with four populations in 2011, suggesting a positive correlation between resistance level and survivorship in the DD bioassays. Biochemical tests conducted with seven insect populations in 2006 and 2015, detected increasing metabolic alterations of all major classes of detoxifying enzymes, mostly of mixed function oxidases. Genotyping of the voltage-gated sodium channel (*AaNa*_*V*_, the pyrethroid target-site) with a TaqMan real time PCR based technique was performed from 2004 to 2014 in all seven localities. The two *kdr* mutations, Val1016Ile and Phe1534Cys, known to be spread throughout Brazil, were always present with a severe decrease of the susceptible allele over time.

**Conclusions/Significance:**

These results are discussed in the context of public and domestic insecticide use, the necessity of implementation of a strong integrated vector control strategy and the conceptual misunderstanding between 'vector control' and 'chemical control of vectors'.

## Author summary

Insecticides will continue to exert an essential role in the reduction of vector density and personal protection for a long time, especially during epidemics. In Brazil, these chemicals are extensively incorporated in national campaigns against *Ae*. *aegypti*, favoring the rise and dispersion of resistant populations. The State of São Paulo (SP), the capital (also named São Paulo) being the 5^th^ most populated city in the world, interrupted pyrethroid application in 2000 given the high resistance levels registered. Nevertheless, SP *Ae*. *aegypti* populations remain resistant to pyrethroids with an ever increasing frequency of *kdr* mutations, one of the main mechanisms responsible for resistance to these chemicals. Although no longer employed by public authorities, pyrethroids are readily available on the commercial market and are extensively used by households as well as private companies, which may partially account for the high resistance ratios still in effect. All in all, it is essential to invest in basic infrastructural sanitation and community educational methods to consciously avoid larval breading sites as well as encourage the correct use of insecticides. Without this, we are fated to forfeit the efficacy of all insecticide classes as well as undermine any other new methods of *Aedes* control.

## Introduction

Latin America has been the epicenter not only of a severe increase in the number of dengue cases, but also of the more recently emerging chikungunya and Zika viruses [1, 2]. The recent cluster of microcephaly in newborns together with other neurological disorders reported in Brazil following a similar scenario in French Polynesia in 2014, has led the World Health Organization (WHO) to declare Zika as a *Public Health Emergency of International Concern* [3, 4]. The lack of specific drugs and effective vaccines against these infections reinforce the importance of controlling their main vector, the *Aedes aegypti* mosquito. Elimination or sealing of artificial containers and water accumulation in domestic and peridomestic surroundings is increasingly considered the most effective way to avoid proliferation of potential larval breeding sites. However, insecticides still play a relevant role in vector control, either as adulticides through space spraying or as larvicides applied in man-made water collections [5]. Unfortunately, the intense use of these insecticides has resulted in the selection of resistant mosquito populations to compounds of several classes [6], hampering *Ae*. *aegypti* control and in consequence, the pathogens transmitted by this vector.

The current goal of public health policy makers is to keep *Ae*. *aegypti* infestation levels below considered risk thresholds [7, 8]. However in the 1950's, *Ae*. *aegypti* erradication was already a challenge with the intense use of organochlorine DDT selecting resistant populations virtually at a worldwide scale [9]. Similarly, resistance to pyrethroids is now widespread throughout the globe [10], mainly due to a serious conceptual misunderstanding between 'vector control' and 'chemical control of vectors' [11]. Ideally insecticide use should be an activity complementary to the mechanical control, the latter based on the removal or elimination of potential breeding sites. Nevertheless, pyrethroids compared to other classes of insecticides are safer for man, bear high insecticidal potency and possess a rapid knock-down effect [12], which contribute to their intense use by both public managers and private initiative. Synthetic pyrethroid resistance dates from the 1990s for *Ae*. *aegypti* populations from US, Caribbean and Asia [9] and has been registered in Brazil since 2000 [13–15]. More recent studies point to increasing resistance levels to pyrethroids in consequence of their use against dengue in endemic areas, especially in Asia, Caribbean and South America [16–19].

Throughout the XX century, Brazil was considered free of *Ae*. *aegypti* twice, in 1958 and in 1973, but this mosquito was detected again in 1967 and later in 1976. However, it was only since 1985 that dengue outbreaks began uninterruptedly in the country [8,20]. In São Paulo (SP), the most urbanized Brazilian State, a novel *Ae*. *aegypti* infestation was detected in 1985, followed by the first local dengue case in its western region, in 1986 [21]. At the beginning of the 1990’s, dengue transmission occurred in the northern SP region quickly expanding to its northwest and coastal regions with high incidence around the seaside city of Santos. High incidence (number of cases/100,000 inhabitants) of dengue was registered in SP between 2006 (150.2) and 2007 (275.9) with transmission expanding toward the central west of the State, reaching 503.0 in 2010. The Brazilian Ministry of Health considers dengue incidence rates above 300 indicative of an epidemic scenario [22]. In 2014 the incidence of dengue was 515.2 in SP disseminating throughout the whole State with distinct incidence patterns [23]. Currently in Brazil besides dengue, chikungunya and Zika viruses are also autochthonously transmitted by *Ae*. *aegypti* [24]. In 2016, SP alone registered 155,972, 3,857 and 232 confirmed autochthonous cases of dengue, Zika and chikungunya, respectively [23].

The Superintendência de Controle de Endemias (Sucen) is the SP division of the Health Secretariat responsible for coordinating insect vector surveillance and control activities in the whole State. Space spraying has been adopted since 1985, applications generally restricted to the summer and autumn in conjunction with the seasonality of both mosquito infestation levels and dengue cases [25]. Several insecticides have been employed against adults in SP, in particular the carbamate propoxur (1986–1989), the organophosphate malathion (1985–1992) and the pyrethroid cypermethrin (1989 to 2000) [26]. In practice, SP State started and stopped pyrethroid application against *Ae*. *aegypti* adults long before the rest of the country. In 1996, Sucen initiated an insecticide resistance surveillance program of *Ae*. *aegypti* populations in SP [27]. This program monitored municipalities subjected to an intense use of insecticides conducted by public health managers due to high dengue incidence [28]. A Brazilian network of *Ae*. *aegypti* insecticide resistance monitoring covering mosquito populations throughout the country was also implemented, in 1999 [29]. In 2000, cypermethrin resistance detection in several *Ae*. *aegypti* populations effected the interruption of pyrethroids and the resumption of malathion in SP. However, the uncontrolled domestic and private use of pyrethroids prevailed by means of ordinary commercial spraying and fogging hired from private agencies.

DDT and pyrethroids induce a characteristic intoxication known as the knockdown effect in which the insect undergoes fast and repetitive muscle spasms followed by paralysis and eventually death [30]. One of the main resistance mechanisms selected against these classes of insecticides is a change in their molecular target, the voltage gated sodium channel (Na_V_). Na_V_ mutations are related to knockdown resistance in several insects, therefore denominated *kdr* mutations, most of which are substitutions of Leu, in the 1014 codon, for Phe, Ser or Hys, as witnessed in *Anopheles* and *Culex* mosquitoes [31, 32]. Due to differences in the codon usage, *kdr* mutations at position 1014 have not been detected in *Ae*. *aegypti*. However, at least seven other punctual mutations have been evidenced worldwide in this mosquito, among which substitutions in the 1016 and 1534 codon sites exhibit the strongest correlation with knockdown resistance [16, 33–35]. In Brazil, there are at least two *kdr* alleles, Na_V_R1 and Na_V_R2. The former has a mutation in the 1534 site of the channel (Phe1534Cys) and the other presents an additional substitution in the 1016 site (Val1016Ile + 1534Cys) [31, 33].

Metabolic resistance is another major physiological mechanism and refers to an increase in the synthesis of detoxifying enzymes or in their specificity to metabolize the insecticide, both resulting in an enhancement of the insect detoxifying capacity [36]. Glutathione S-transferases (GST), carboxylesterases (EST) and multi-function oxidases (MFO, also known as P450) are the key classes of enzymes generally enrolled in this process, all belonging to families composed of several genes [11, 36]. Some of these enzymes potentially act over a broad nature of xenobiotics, resulting in cross-resistance among different classes of insecticides [37, 38]. In addition to these generic activities, changes in some genes can be selected to detoxify a specific compound [39]. Among these detoxifying enzymes, the MFO class deserves attention in resistance of *Ae*. *aegypti* to pyrethroids, as revealed by high-throughput assays, by comparing the overall profile at genomic and transcriptome levels between resistant and susceptible populations [40–43].

Insecticide bioassays are the primary methods for monitoring the resistance status of natural populations [44]. Further investigation of selected mechanisms may anticipate the presence of resistance alleles or specific activities before resistance reaches critical levels in a population. Based on these data, surveillance is likely to guide control actions more effectively and locally oriented. We show the temporal analysis of the pyrethroid susceptibility status of several *Ae*. *aegypti* populations from SP State along with the profiles of the main potentially related mechanisms, target site and metabolic resistance. Our aim is to describe this resistance scenario since the history of insecticide utilization in SP is different from the rest of the country, as stated above. If on one hand dengue incidence depends on many factors such as mosquito abundance, immune population frequency and vector competence of mosquitoes, on the other hand, dengue outbreaks can often lead to an uncontrolled increase in the domestic use of insecticides, available in the retail marked. The intensity of insecticide applications by governmental campaigns as well as the dynamics of *Ae*. *aegypti* resistance status in SP have been well monitored [15, 26, 45]. We analysed data related to pyrethroid resistance status, enzymatic profiles of detoxifying enzymes and the frequency of *kdr* alleles in *Ae*. *aegypti* populations from strategic areas of SP under a time series perspective. Although based on a regional scale, conclusions of this study may contribute to unfold the phenomena of insecticide resistance and help to design sustainable vector control strategies.

## Materials and methods

### Sampling

In SP, the insecticide resistance of *Ae*. *aegypti* populations from 16 municipalities is regularly monitored. In this study we focused the analysis on seven of them with different profiles of dengue incidence, an indirect indicator of insecticide use intensity. As representatives of their respective regions, *Ae*. *aegypti* samples of discriminated municipalities are listed in descending order of dengue cumulative incidence from 1998 to 2014 (cases per 100,000 inhabitants): Sao José do Rio Preto (20,203), Araçatuba (14,779), Santos (14,010), Ribeirão Preto (9,346), Campinas (5,800), Presidente Prudente (2,446) and Marília (2,084). Field sampling was performed yearly with ovitraps as described elsewhere [46] from November to December, which corresponds to the pre-epidemic season. The Rockefeller strain (Rock), continually maintained in the laboratory, was always referred to in parallel both as an insecticide susceptibility control and an internal control of laboratory assays [47].

### Bioassays

Although cypermethrin was employed in SP from 1989 to 2001, in 2000 deltamethirn was implemented in the whole country, except SP. However, as the insecticide resistance monitoring evaluations were conducted at a national level, deltamethrin was the compound of choice for evaluating adult resistance to pyrethroids. The status of susceptibility/resistance to the pyrethroid deltamethrin was evaluated by dose-diagnostic (DD) mortality bioassays with impregnated papers and the WHO test tube kits according to the WHO guidelines [48, 49]. Papers were impregnated in the laboratory with the recommended diagnostic dosage of 0.05% (18 mg/a.i/m^2^). Around 20 non-blood fed 2–5 day old adult females were exposed during 1 hour to deltamethrin and then transferred to the resting tube free of insecticide where mortality was recorded 24 hours later. The assays for each population were performed in triplicate and were repeated at least three times with F1 or F2 females, average mortality below 80% indicating resistance, according to the WHO criteria in force at the time of the assays [48, 49]. In addition to DD tests, a dose-response (DR) assay was also performed with F1 or F2 generations of four of the populations collected in 2011. DR procedures were similar to the DD test and incorporated four to seven different deltamethrin concentrations. Data were submitted to Probit transformation followed by linear regression analysis with the help of the software Polo PC [50] in order to calculate the lethal doses (LD).

### Biochemical assays

The activity of esterases (with both substrates α– and β–naphthyl acetates), multi-function oxidases (MFO) and glutathione-S-transferases (GST) enzymes was quantified in samples of around 30 individual adult females one-day post-emergence. These tests were carried out according to adaptations to the instructions from the Centers for Disease Control (CDC) and WHO [51, 52].

### *kdr* genotyping

DNA was extracted from each of the abdomen deprived females which were tittered in 200 μL of “squishing buffer” (10 mM Tris-HCl pH 8.2, 2 mM EDTA and 0.2% Triton X-100) according to Jowett (1986), followed by an incubation with 0.2 mg/L proteinase K (Promega) at 56°C overnight and an inactivation final step at 95°C for 5 minutes. Both 1016 (Val^+^ and Ile^*kdr*^) and 1534 (Phe^+^ and Cys^*kdr*^) sites of the *Ae*. *aegypti* Na_V_ were genotyped with a customized TaqMan genotyping assay (Thermo Fischer Scientific) independently for each site. The sequences of primers and probes are available in Table 1. The reactions were conducted in a 10 μL mix containing 1 μL of DNA (~10 ng), 1 X TaqMan Genotyping Master-mix and the TaqMan assay combined primers and probes 1X for 1016 assay or 0.5 X for 1534 assay. Around 30 specimens of each population were individually evaluated in a 96 well microplate together with the positive controls: SS (Rock strain), RR (Rock-*kdr* strain, [53]) and RS (an equimolar mix or Rock and Rock-kdr). The thermocycling program was in accordance with the manual instructions (TaqMan genotyping assay, Thermo Fischer Scientific) in StepOne Plus or 7500 real time machines (Thermo Fischer Scientific). As previously described [33], allelic and genotypic frequencies took into account that the 1016 and 1534 SNPs are placed in a single locus, constituted by the alleles: Na_V_S (1016 Val^+^ + 1534 Phe^+^), Na_V_R1 (1016 Val^+^ + 1534 Cys^*kdr*^) and Na_V_R2 (1016 Ile^*kdr*^ + 1534 Cys^*kdr*^). Table 2 evidences how each genotype was obtained based on the result of each SNP. Hardy-Weinberg equilibrium was assessed by the classical equation [54], the null hypothesis of equilibrium checked by a chi-square test with one or three degrees of freedom when three or six genotypes, respectively, were evidenced.

**Table 1:** Sequences of primers and probes used in the genotyping reactions for 1016 and 1534 Na_V_ sites of *Aedes aegypti*.

1016	primer forward	CGTGCTAACCGACAAATTGTTTCC
	primer reverse	GACAAAAGCAAGGCTAAGAAAAGGT
	probe Val^+^	VIC-CCGCACAGATACTTA-NFQ
	probe Ile^*kdr*^	FAM-CCCGCACAGGTACTTA-NFQ
1534	primer forward	CGAGACCAACATCTACATGTACCT
	primer reverse	GATGATGACACCGATGAACAGATTC
	probe Phe^+^	FAM-ACGACCCGAAGATGA-NFQ
	probe Cys^*kdr*^	VIC-AACGACCCGCAGATGA-NFQ

All sequences are oriented in 5’-3’ sense. FAM and VIC refer to the reporter dye and NFQ to the quencher. The manufactures’ IDs for the sites 1016 and 1534 are respectively AHS1DL6 and AHUADFA.

**Table 2:** Possible genotypes for Latin American *Aedes aegypti* populations, considering the single nucleotide polymorphisms in the 1016 and 1534 Na_V_ sites.

SNPs		Genotypes	Allelic composition
1016	1534		
Val/Val	Phe/Phe	**SS**	Na_V_S/Na_V_S
	Phe/Cys	**SR1**	Na_V_S/Na_V_R1
	Cys/Cys	**R1R1**	Na_V_R1/Na_V_R1
Val/Ile	Phe/Phe	SR3	
	Phe/Cys	**SR2** +R1R3	Na_V_S/Na_V_R2
	Cys/Cys	**R1R2**	Na_V_R1/Na_V_R2
Ile/Ile	Phe/Phe	R3R3	
	Phe/Cys	R2R3	
	Cys/Cys	**R2R2**	Na_V_R2/Na_V_R2

The alleles were determined based on the observed genotypes, merging the 1016 and 1534 SNP reactions. As there was no evidence of any insect genotyped as SR3, R2R3 and R3R3 in this work and elsewhere [33, 58], summed with the unlikely existence of the Na_V_R3 allele [71], we considered all 1016 (Val/Ile) + 1534 (Phe/Cys) individuals as SR2.

## Results

### Bioassays

*Aedes aegypti* samples of seven SP municipalities, field-collected every year from 2006 to 2014, were exposed to DD bioassays with the pyrethroid deltamethrin (Table 3). In this period the criteria for interpreting bioassays with diagnostic dose classified all populations as resistant, except Campinas in 2013 and Marília in 2009 as their mortality rates exceeded 80% [48]. According to the current criterion, however, all the analysed populations would have been considered resistant to deltamethrin [55, 56]. In principle, DD tests are qualitative, i.e. useful only for classification of a population as resistant or susceptible. In addition, quantitative DR deltamethrin assays were performed with four populations collected in 2011 (Table 4), confirming the resistance status previously detected by the DD assays. Notwithstanding, we found a significant correlation between DD mortality levels and DR derived resistance ratios (RR) in the four populations submitted to both assays. In other words, the higher the RR of a population, the more specimens survived the diagnostic dose. This correlation served for both RR_50_ (R^2^ = 0.9485, p = 0.0261) and RR_95_ (R^2^ = 0.9399, p = 0.0305), suggesting that some quantitative character could be attributed in this case, comparatively, to the DD assays.

**Table 3:** Qualitative (DD) deltamethrin bioassays with F1 or F2 *Aedes aegypti* female adults derived from São Paulo State populations collected from 2006 to 2014.

Locality	2006		2007		2008		2009		2010		2011		2012		2013		2014	
Araçatuba	45.1	(18.3)	26.6	(14.1)	34.0	(13.0)	38.1	(9.8)	42.5	(22.5)	64.3	(20.7)	48.2	(10.1)	49.3	(11.0)	12.7	(6.8)
Campinas	-		56.3	(9.8)	34.0	(13.0)	53.6	(12.6)	-		65.5	(14.1)	69.9	(3.8)	80.2	(2.9)	51.2	(13.2)
Marília	-		68.8	(7.3)	-		81.3	(7.4)	-		73.5	(12.0)	65.6	(13.7)	57.8	(15.5)	57.5	(12.6)
P Prudente	53.9	(26.2)	49.1	(17.6)	32.7	(12.3)	46.0	(8.8)	59.4	(9.3)	40.5	(20.5)	73.3	(6.7)	44.5	(21.9)	61.7	(23.1)
Ribeirao Preto	38.8	(22.8)	39.0	(17.2)	24.1	(19.4)	49.1	(17.9)	55.1	(17.8)	69.7	(8.9)	48.6	(12.2)	46.5	(21.0)	51.0	(6.3)
SJ R Preto	65.6	(7.6)	57.0	(28.6)	-		47.4	(15.2)	58.8	(20.9)	-		51.8	(14.2)	36.3	(12.3)	44.7	(21.3)
Santos	25.4	(12.0)	24.3	(20.0)	-		58	(20.1)	50.5	(13.4)	49.6	(5.8)	28.3	(7.3)	36.8	(8.7)	40.4	(15.6)

Numbers refer to the mortality rates with SD inside parenthesis.

(-) non evaluated.

**Table 4:** Quantitative (DR) deltamethrin bioassays with *Aedes aegypti* female adults derived from São Paulo State populations collected in 2011.

Population/ strain	Mortality parameters*					Resistance ratios	
	LD_50_		LD_95_		slope	RR_50_	RR_95_
**Rockefeller**	0.5	(0.5–0.6)	1.5	(1.3–1.8)	3.6	1.0	1.0
**Marília**	8.7	(7.4–10.1)	59.8	(48.0–78.0)	2.0	17.1	40.4
**Ribeirão Preto**	11.6	(8.4–14.9)	86.2	(60.0–114.1)	1.9	22.7	58.2
**Araçatuba**	19.5	(17.0–22.0)	107.0	(88.0–136.4)	2.2	38.2	72.3
**Santos**	27.3	(25.3–29.4)	140.3	(118.7–172.4)	2.3	53.5	94.8

*Lethal doses for 50% (LD_50_) and 95% (LD_95_) of a population (together with its respective 95% confidence intervals). The linear regression slope is an estimation of the population heterogeneity.

**Fig 1:**
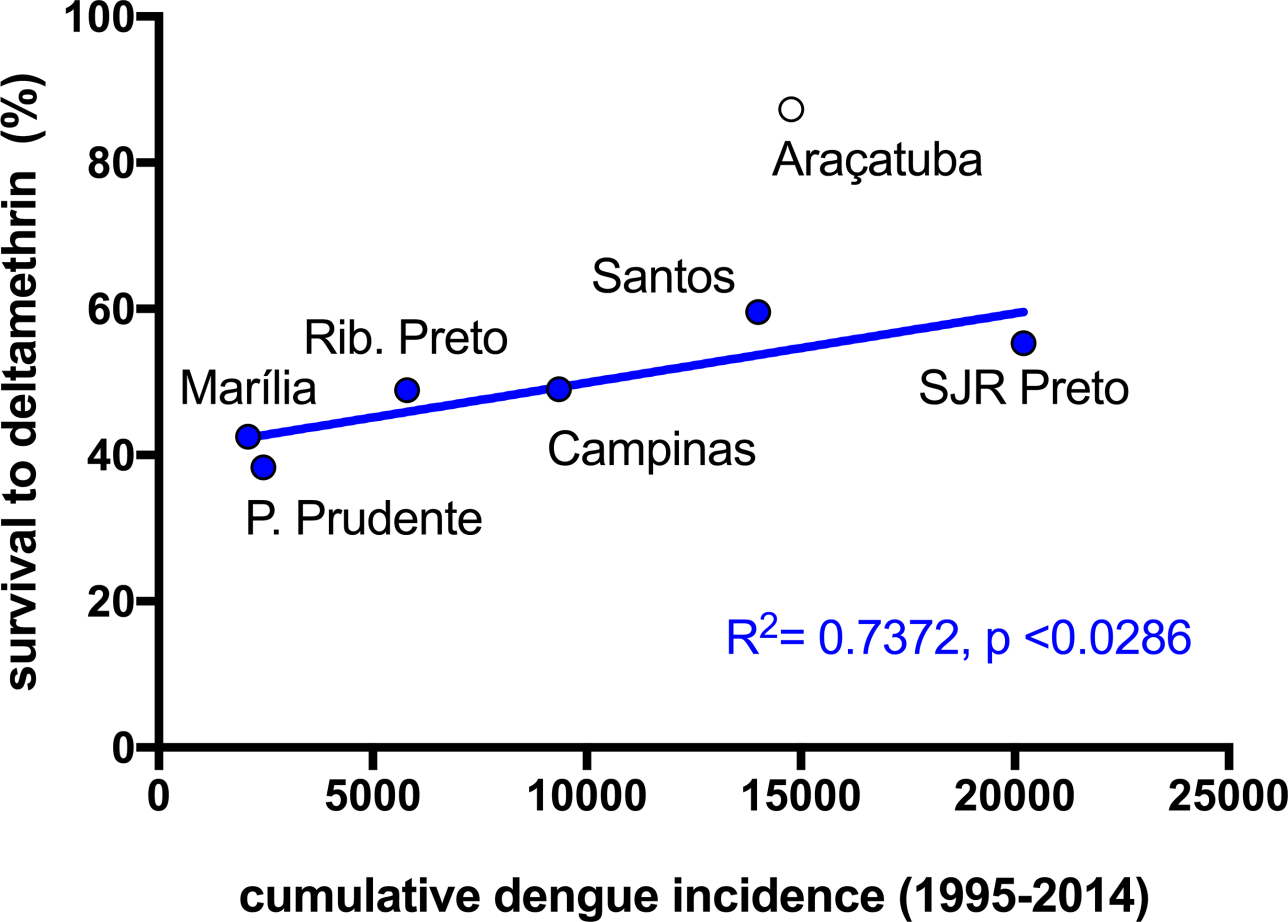
Does the intensification of domestic insecticide use due to dengue outbreaks may have an impact on *Ae*. *aegypti* resistance to pyrethroids? Correlation analysis between the cumulative incidence of dengue cases (1995–2014) in six localities of São Paulo State and *Aedes aegypti* survival after exposure to the deltamethrin qualitative dose-diagnostic bioassay of samples collected in 2014. Araçatuba was plotted in the figure but not considered in the correlation analysis (see text for details).

Due to the explosive aspect of dengue epidemics, it is possible to observe a significant intensification of the domestic use of insecticides, usually pyrethroids, during outbreaks. We investigated, indirectly, whether this indiscriminate chemical control could impact resistance to pyrethroids: dengue incidence rates were plotted against the results obtained with the bioassays with the diagnostic dose of deltamethrin. We found a positive correlation between the cumulative dengue incidence (number of cases/ 100,000 inhabitants) from 1995 to 2014 and survival to the deltamethrin DD in 2014 (R^2^ = 0.4196, p = 0.1157). This correlation increases (R^2^ = 0.7372, p = 0.0286) if we exclude Araçatuba from the analysis (Fig 1), considering that the mortality index in DD tests for this locality in 2014 substantially differed from its time series and other localities that year (Table 3).

### *Kdr* genotyping

**Fig 2:**
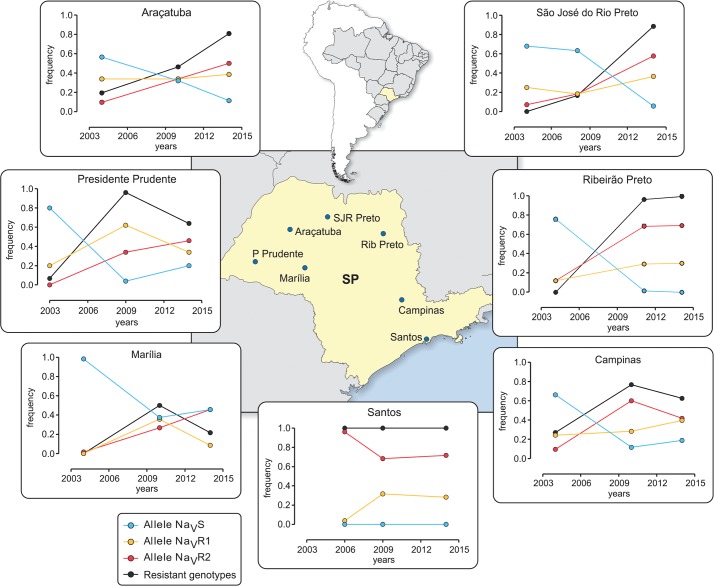
Frequencies of susceptible (Na_V_S) or *kdr* (Na_V_R1 and Na_V_R2) alleles and resistance genotypes (R1R1, R1/R2 and R2R2) in *Aedes aegypti* populations from São Paulo State (SP) between 2003 and 2014. The composition of each allele considered both 1016 and 1534 Na_V_ sites for each genotyped individual: Na_V_S (1016 Val^+^ + 1534 Phe^+^), Na_V_R1 (1016 Val^+^ + 1534 Cys^*kdr*^) and Na_V_R2 (1016 Ile^*kdr*^ + 1534 Cys^*kdr*^). The municipalities are placed inside the highlighted map of SP State.

*Kdr* genotyping was carried out with around 30 specimens of the seven SP localities exposed to DD, as mentioned above. In all cases, three samples were employed, collected from 2003 to 2014. The allelic composition considered the result of both genotyping reactions (for 1016 and 1534 Na_V_ sites) for each insect, herein referred to as Na_V_S (1016 Val^+^ + 1534 Phe^+^), Na_V_R1 (1016 Val^+^ + 1534 Cys^*kdr*^) and Na_V_R2 (1016 Ile^*kdr*^ + 1534 Cys^*kdr*^). The theoretically possible allele Na_V_R3 (1016 Ile^*kdr*^ + 1534 Phe^+^) was not detected. Fig 2 displays the variations in the frequencies of each Na_V_ allele and the pooled “Resistant” genotypes, indicating their respective localities in the map. Overall, there was an increase in the frequencies of the resistant *kdr* alleles. In this scenario, considering the difference between final and initial frequencies, the minimal observed decline of the Na_V_S allele in the period was 45% in Araçatuba, the greatest difference, 76%, noted in Ribeirão Preto. One must be aware that the Na_V_S allele was not detected in Santos since its first sampling, in 2006. Although both Na_V_R1 and Na_V_R2 *kdr* alleles increased in the period with differing dynamics, the latter exhibited a greater rise, lowest and highest increase being 32% (Campinas) and 58% (Ribeirão Preto), respectively. In contrast, the lowest and highest increase of Na_V_R1 were 5% and 30%, respectively, in Araçatuba and Santos (S1 Table).

Considering that knockdown resistance based on the *kdr* mutations is a recessive trait, individuals with the genotypes R1R1, R1R2 and R2R2 should potentially represent the pyrethroid resistant portion of the population for this target-site mechanism at least. In this case, for the most recent sampling (2014), Marília presented the lowest frequency of resistant genotypes (22%), in contrast with 100% observed in Santos and Ribeirão Preto (Table 5). All individuals from Santos were genotyped as”resistant” in the three sampling times. Meanwhile, Ribeirão Preto mosquitoes exhibited the highest rise in the resistant genotypes, from 0 in 2004 to 100% a decade later. The decrease of the S allele in all populations between 2004 and 2014 can be seen in S1 Fig.

**Table 5:** *Kdr* genotypes in *Aedes aegypti* populations from São Paulo State from 2004 to 2014.

Locality	sampling year	n*	Genotype frequencies							
			SS	SR1	R1R1	SR2	R1R2	R2R2	Susceptible**	Resistant**
Araçatuba	2004	31	0.323	0.387	0.129	0.097	0.032	0.032	0.806	0.194
	2010	28	0.107	0.214	0.214	0.214	0.036	0.214	0.536	0.464
	2014	26	0.038	0	0.154	0.115	0.423	0.231	0.192	0.808
Campinas	2004	37	0.595	0.108	0.189	0.027	0	0.081	0.730	0.270
	2010	30	0	0.033	0.233	0.200	0.067	0.467	0.233	0.767
	2014	24	0	0.125	0.167	0.250	0.333	0.125	0.375	0.625
Marília	2004	29	0.966	0	0	0.034	0	0	1	0
	2010	28	0.250	0.143	0.107	0.107	0.357	0.036	0.500	0.500
	2014	23	0.130	0.043	0.043	0.609	0	0.130	0.783	0.217
Presidente Prudente	2003	30	0.667	0.267	0.067	0	0	0	0.933	0.067
	2009	25	0.040	0	0.440	0	0.360	0.160	0.040	0.960
	2014	25	0.040	0.040	0.120	0.280	0.400	0.120	0.360	0.640
Ribeirão Preto	2004	25	0.520	0.240	0	0.240	0	0	1	0
	2011	29	0	0.034	0.103	0	0.345	0.517	0.034	0.966
	2014	28	0	0	0.071	0	0.464	0.464	0	1
Santos	2006	26	0	0	0.038	0	0	0.962	0	1
	2009	30	0	0	0.133	0	0.367	0.500	0	1
	2014	34	0	0	0.118	0	0.441	0.441	0	1
São José do Rio Preto	2004	30	0.357	0.500	0	0.143	0	0	1	0
	2008	26	0.433	0.233	0.033	0.167	0.067	0.067	0.833	0.167
	2014	28	0	0	0.077	0	0.577	0.231	0	0.885

The genotypes considered *Aedes aegypti* Na_V_ at both positions 1016 and 1534 for the alleles S (1016 Val^+^ + 1534 Phe^+^), R1 (1016 Val^+^ + 1534 Cys^*kdr*^) and R2 (1016 Ile^*kdr*^ + 1534 Cys^*kdr*^).

*number of specimens

**Genotypes containing at least one S allele were considered susceptible.

### Biochemical assays

**Fig 3:**
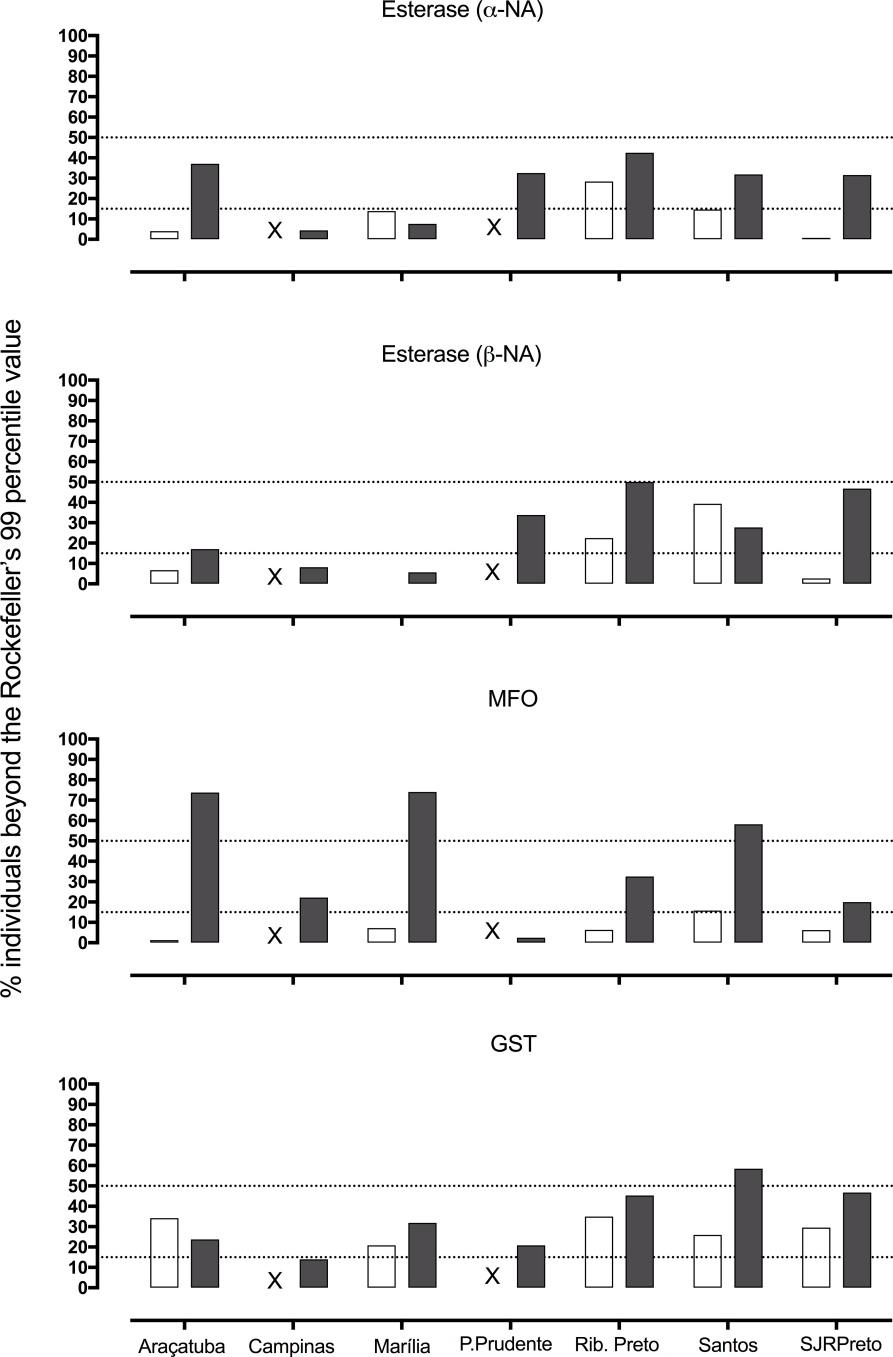
Evaluation of enzymes related to metabolic resistance in *Aedes aegypti* populations from SP State in two moments from 2006 to 2015. The bars represent the enzymatic activity in % beyond the 99 percentile of Rockefeller lineage, run in parallel (white bars = 2006 and grey bars = 2015). The values beyond 15 and 50%, indicated by the dotted lines, were respectively classified as altered or highly altered. The “X” mark indicates samples not evaluated.

Alterations in the activity of the main detoxifying enzyme classes were investigated in *Ae*. *aegypti* from all SP municipalities evaluated, collected both in 2006 and 2015 (Fig 3), as an indication for the selection of metabolic resistance mechanisms. In general, an increase in Esterases, MFO and GSTs activities was evidenced during the study time. The activities were considered either ‘altered’ or ‘highly altered’ if more than 15% or 50%, respectively, of the population presented values beyond the Rockefeller’s 99 percentile [51]. In this sense with the exception of Campinas and Marília, the remaining populations were classified as ‘altered’ for Esterases, evaluated with both αNA and βNA substrates. Araçatuba, Marília and Santos were ‘highly altered’ for MFO, presenting a substantial increase compared to the previous evaluation. Excluding Campinas, GST was ‘altered’ in all assessments and Santos (2015) ‘highly altered’.

## Discussion

The susceptibility status of *Aedes aegypti* to insecticides has been annually monitored in SP State since 1996 by the Superintendência de Controle de Endemias (SUCEN) and accompanied as an integrant of the national network (MoReNAa Network) since 1999 [29]. Organophosphate compounds have been adopted in Brazil against larvae and adults of *Ae*. *aegypti* since the first contemporary dengue epidemic outbreak in the 1980’s. São Paulo State started employing pyrethroids in governmental campaigns targeting *Ae*. *aegypti* adults in 1989, at least ten years prior to the rest of the country. Ultralow volume (ULV) assays with *Ae*. *aegypti* populations from SP performed in 2000/2001, and afterwards between 2007 and 2009, however, revealed lack of efficacy of the pyrethroids but not of the organophosphate malathion. Based on this scenario, malathion was resumed in the State, replacing the pyrethroids [26]. Again, this change in the rest of the country started later, in 2009 [57], given the broader evidence of disseminated of pyrethroid resistance in Brazilian *Ae*. *aegypti* populations [14, 33, 58]. In this sense, laboratory bioassays have been very useful to the surveillance programs since resistance detected in these tests are correlated with failure of field control operations [18, 26]. Additional semi-field trials of residual treatment indicated lack of efficiency of cypermethrin, malathion, bendiocarb and fenitrothion in Santos but not in Marilia (2002–2003). Afterwards in 2011, this same kind of test displayed ineffectiveness of fenitrothion, deltamethrin and bendiocarb for Marilia, Araçatuba and Santos vector populations [26].

Although pyrethroids have no longer been used for several years in governmental campaigns against *Aedes* in SP, all vector populations here evaluated with bioassays remained resistant to deltamethrin. Araçatuba sampled in 2014 exhibited the lowest mortality level after exposure to this pyrethroid, suggesting that mosquitoes from that locality could substantially differ from the others. However, a previous population genetics study with *Ae*. *aegypti* populations from SP did not provide any evidence of an unusual genetic difference of Araçatuba insects [59]. In that study, Araçatuba, Presidente Prudente and Marília were grouped in a clade. On the other hand, insecticide residual spraying (IRS) with pyrethroids occurs since 1999 in Araçatuba, due to cases of urban visceral leishmaniosis [60]. This intensified use of pyrethroids probably contributed to the special low mortality rates of Araçatuba mosquitoes in deltamethrin bioassays, when compared to other SP localities and reinforce the need for integrated vector control methods. The increase in the frequencies of *kdr* alleles in all other localities suggests that a selection pressure with pyrethroids still persists. Although hard to precisely quantify, an increase in the use of household sprays, all of them formulated with pyrethroids, was noted. A tendency of intensification of metabolic resistance was also observed. However, this could also be attributed to organophosphate pressure [10]. Further bioassays with organophosphates will help us to better address this hypothesis. The lack of a well-structured integrated vector control strategy with emphasis on mechanical control and community engagement towards the prevention and elimination of breeding sites, is needed. In this sense, chemical control should be envisaged as a complementary approach regarding vector control activities, and, ideally, insecticide adoption should take into account the potential overlap of different synantropic vector species.

Herein, we followed the dynamics of the Na_V_ alleles during a decade in seven *Ae*. *aegypti* populations of SP. The three alleles, Na_V_S, Na_V_R1 and Na_V_R2 previously identified in Brazilian populations [33, 61], were confirmed. As *kdr* mutations are physiologically expressed as a recessive trait, only the *kdr* homozygous insects are potentially resistant to pyrethroids [32]. Mutant alleles can remain at low levels for a long period and therefore, resistance based on this mechanism may delay to be established in the field. This happens because initially most of the mutant alleles are carried by heterozygotes, which are not favourably selected by insecticide exposure. On the other hand under similar conditions, as *kdr* homozygotes appear, these alleles can spread very rapidly [62]. For instance, in the present study at Ribeirão Preto, no Na_V_R1 or Na_V_R2 *kdr* homozygote was found in 2004. However 48% of the population presented one of these alleles as heterozygotes, meaning that none of them would be potentially resistant to pyrethroids through this target site mechanism. In 2014, 100% of genotyped *Ae*. *aegypti* from Ribeirão Preto presented a genotype with the potential for resistance (R1R1, R1R2 and R2R2).

Besides genotyping of known molecular markers for resistance, additional approaches are unraveling other factors enrolled in the physiology of metabolic resistance mechanisms. This is the case of genomic and transcriptomic analysis, based on microarrays developed to specifically assess detox genes, and high-throughput sequencing with resistant populations of *Ae*. *aegypti*. Beyond mutations on protein coding sequences, copy gene amplifications have been identified in detox genes as polymorphisms associated with pyrethroid resistance in *Ae*. *aegypti* [40, 63, 64]. These mechanisms are so dynamic and variable among populations that identifying a specific gene or a mutation as a diagnostic marker for resistance is a difficult task [65].

The so called 'biochemical tests' in turn only indicate general activity alterations of broad classes of enzymes from a test population compared to a reference lineage [66]. Based on this method, an increase in the activity of Esterases and GSTs was evident in *Ae*. *aegypti* populations from Brazil between 2001 and 2004 [52]. Herein, we also observed a great change in the MFO profile. Accordingly, the key participation of *Cyp450* genes is detected by genomic and transcriptomic studies. For instance, three unrelated deltamethrin resistant *Ae*. *aegypti* populations from French Guiana (South America), Guadaloupe islands (Lesser Antilles) and New Caledonia (Pacific Ocean) shared alterations in four out of five common up-regulated *Cyp450* genes [16]. Although it is not easy to trace linear correlations between enzymatic activity and mortality values, some general relationships can be considered. For instance, Santos and Ribeirão Preto presented the most altered metabolic resistance profiles in 2015 related to 2006 (Fig 3). These were also the localities where 100% of screened insects presented a *kdr* ‘R genotypes’ in 2014 (Table 5).

Failure in controlling vector population densities ultimately results in high incidence of the diseases derived from the arboviruses/parasites they transmit. There are several operational reasons for this flaw, insecticide resistance being an important issue when insecticides are the major tool towards vector control [10, 67]. It is not uncommon that failure of vector control with insecticides triggers the use of even more insecticides, leading to resistance increase and dissemination. When an epidemic situation is established this vicious cycle can reach extremely high levels. This is a consequence, among others, of the great increase in the domestic use of insecticides, motivated by the population's panic. This situation has been recently reported in different Brazilian municipalities [68]. The overall positive correlation found in SP State municipalities between cumulative dengue case incidence and survival levels of *Ae*. *aegypti* in the pyrethroid bioassays is an indirect evidence of this phenomenon: the greater the incidence of dengue, the more intense 'individual' attempts to control its vector; in this case with the use of insecticides for which the resistance is already installed. The consequence is evident, that is, more resistance increase. This indirect correlation, together with data demonstrating lack of insecticide efficacy in the field when laboratory bioassays had indicated loss of susceptibility [26], corroborates the importance of insecticide resistance monitoring programs, especially when the results are considered for vector control strategies.

Currently the Brazilian Dengue Control Program indicates the use of Insect Growth Regulators against larvae and the organophosphate malathion for adults in *Ae*. *aegypti* control campaigns [57], where no resistance has yet been reported in the country, except for some very specific localities. The continuous susceptibility status surveillance of field populations based on bioassays is important to evaluate the efficiency of regularly employed insecticides as well as the potential alternatives. Dose-response assays were performed in 2011, aiming to evaluate whether any quantitative character in terms of resistance level could be inferred from the dose-diagnostic tests, in principle a qualitative assay, that classifies populations as susceptible or resistant. We witnessed a significant correlation among resistance ratios (obtained from dose-response assays) and mortality values (from dose-diagnostic assays) based on the four populations assessed in both tests. Field response was also in line with the diagnostic-dose assay, where pyrethroid ULV field test applications resulted in failure for populations classified as resistant through laboratory bioassays [26]. The São Paulo State monitoring program uses only diagnostic-dose testing since it is more rapid and requires fewer insects and lower amounts of impregnated papers compared to the quantitative assays. Additionally, in the case of *kdr* mutations, genotyping of molecular markers for resistance to pyrethroids can predict the dynamics of the genetic background of a given population before resistance is established. However, when the frequency of the *kdr* allele exceeds the “tipping-point” and selection pressure persists in the environment, the resistant status is very rapidly achieved in the population [69,70].

### Conclusions

Although pyrethroids have no longer been administrated by public health campaigns against *Ae*. *aegypti* in SP State since 2001, the mosquito is still resistant to this class of insecticide. Both the frequency of *kdr* mutations and the altered activity of detoxifying enzymes have been increasing. High dengue incidence rates in the successive epidemics throughout the country, along with a strong bias towards chemical control, are probably the main reasons motivating people to utilize excessive amounts of domestic insecticides which are mostly pyrethroids. Therefore, the selection pressure with pyrethroids continues to be relevant. Also, the insecticide based strategies for controlling other vector species, such as *Culex*, *Anopheles*, triatomines and sandflies, should be integrated considering insecticide resistance peculiarities and management of each species. Measures taken by the public health managers to avoid increase and spread of insecticide resistance and cross-resistance should also consider domestic use. The most efficient manner for vector control of dengue, chikungunya and Zika is undoubtedly avoiding accumulated water in domestic and urban containers. Nevertheless, chemical control still inspires general overconfidence in society. Community awareness regarding the consequences and limitations of the chemical strategy for controlling *Ae*. *aegypti* and towards the benefits of vector prevention measures is therefore crucial. An integrated vector control strategy is imperative for the optimal use of resources and significant amelioration in the sustainability of chemical control, not only of *Aedes* but also other insect vectors.

## Supporting information

S1 Table Variation between initial and final frequencies of *Aedes aegypti* Na_V_ alleles from São Paulo populations. (DOCX)

S1 Fig Frequencies of the susceptible Na_V_ allele (S: 1016 Val^+^ + 1534 Phe^+^) of *Aedes aegypti* populations from different municipalities of São Paulo State between 2004 and 2014. Beyond the data points, regression lines are also exhibited.
(TIFF)
