# Supplementary material for: Pyrethroid resistance persists after ten years without usage against Aedes aegypti in governmental campaigns: Lessons from São Paulo State, Brazil
Source: PLoS Negl Trop Dis. 2018 Mar 30;12(3):e0006390. doi: 10.1371/journal.pntd.0006390 (PMC5895049; doi:10.1371/journal.pntd.0006390)
Supplement: S1 Table — (DOCX) [file pntd.0006390.s001.docx]

**Supplementary Table S1** – Variation between initial and final frequencies of *Aedes aegypti* Na_V_ alleles from São Paulo populations

| **Locality** | **time period** | **Differences between last and former frequencies evaluated** | | |
| --- | --- | --- | --- | --- |
|  |  | Na_V_S | Na_V_R1 | Na_V_R2 |
| Araçatuba | 2004-2014 | -44.9 | 4.6 | 40.3 |
| Campinas | 2004-2014 | -47.5 | 15.3 | 32.2 |
| Marília | 2004-2014 | -52.6 | 8.7 | 43.9 |
| P.Prudente | 2003-2014 | -60.0 | 14.0 | 46.0 |
| Rib. Preto | 2004-2014 | -76.0 | 18.4 | 57.6 |
| Santos | 2006-2014 | 0 | 30.0 | -30.0 |
| SJ do Rio Preto | 2004-2014 | -62.1 | 11.5 | 50.5 |
