# Supplementary figures and images for: Pyrethroid resistance persists after ten years without usage against Aedes aegypti in governmental campaigns: Lessons from São Paulo State, Brazil
Source: PLoS Negl Trop Dis. 2018 Mar 30;12(3):e0006390. doi: 10.1371/journal.pntd.0006390 (PMC5895049; doi:10.1371/journal.pntd.0006390)

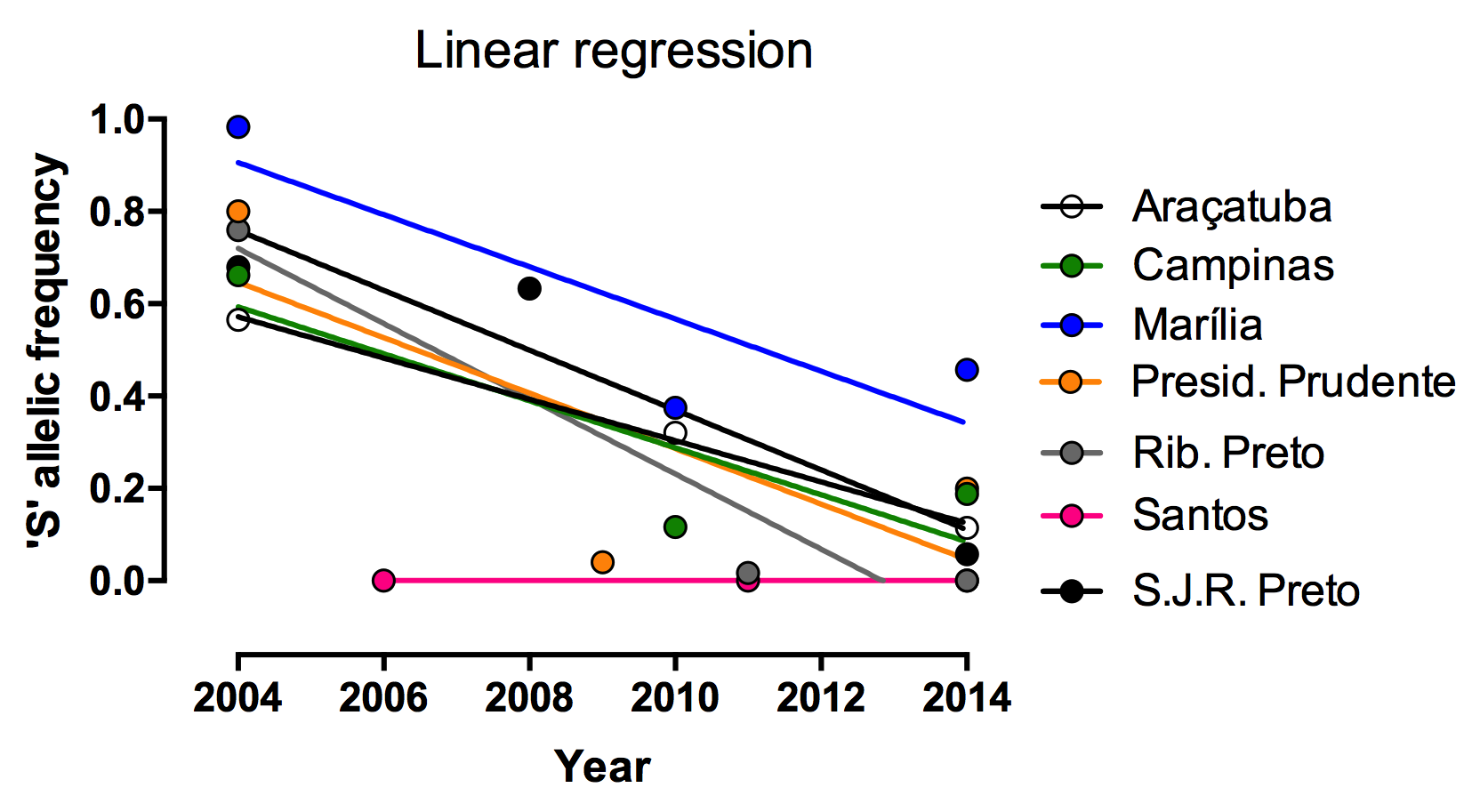

Supplement: S1 Fig — Beyond the data points, regression lines are also exhibited. (TIFF) [file pntd.0006390.s002.tiff]
